# RETRACTION: miR‐709 Up‐Regulated in Hepatocellular Carcinoma, Promotes Proliferation and Invasion by Targeting GPC5

**DOI:** 10.1111/cpr.70151

**Published:** 2025-12-02

## Abstract

**RETRACTION**: LiuT. , ZhangX. , ShaK. , LiuX. , ZhangL. and WangB. , “miR‐709 Up‐Regulated in Hepatocellular Carcinoma, Promotes Proliferation and Invasion by Targeting GPC5,” Cell Proliferation 48, no. 3 (2015): 330–337, 10.1111/cpr.12181.25818666 PMC6495981

The above article, published online on 27 March 2015 in Wiley Online Library (wileyonlinelibrary.com), has been retracted by agreement between the journal Editor‐in‐Chief, Qi Zhou; and John Wiley & Sons Ltd. The retraction has been agreed upon following the identification of duplicated elements between Figures 5c and 7c, which were reported to represent different experimental conditions. The authors provided some supporting data; however, it was insufficient to fully address the concerns. As a result, the editors have lost confidence in the reliability of the results. The authors did not respond when asked to agree to the final wording of the retraction.

**RETRACTION**: T. Liu , X. Zhang , K. Sha , X. Liu , L. Zhang and B. Wang , “miR‐709 Up‐Regulated in Hepatocellular Carcinoma, Promotes Proliferation and Invasion by Targeting GPC5,” Cell Proliferation 48, no. 3 (2015): 330–337, 10.1111/cpr.12181.25818666 PMC6495981

The above article, published online on 27 March 2015 in Wiley Online Library (wileyonlinelibrary.com), has been retracted by agreement between the journal Editor‐in‐Chief, Qi Zhou; and John Wiley & Sons Ltd. The retraction has been agreed upon following the identification of duplicated elements between Figures 5c and 7c, which were reported to represent different experimental conditions. The authors provided some supporting data; however, it was insufficient to fully address the concerns. As a result, the editors have lost confidence in the reliability of the results. The authors did not respond when asked to agree to the final wording of the retraction.

